# Polarization-controllable Airy beams generated via a photoaligned director-variant liquid crystal mask

**DOI:** 10.1038/srep17484

**Published:** 2015-12-02

**Authors:** Bing-Yan Wei, Peng Chen, Wei Hu, Wei Ji, Li-Yang Zheng, Shi-Jun Ge, Yang Ming, Vladimir Chigrinov, Yan-Qing Lu

**Affiliations:** 1 National Laboratory of Solid State Microstructures, Collaborative Innovation Center of Advanced Microstructures and College of Engineering and Applied Sciences, Nanjing University, Nanjing 210093, China; 2 Center for Display Research, Department of Electronic and Computer Engineering, Hong Kong University of Science and Technology, Clear Water Bay, Kowloon, Hong Kong, China

## Abstract

Researches on Airy beams have grown explosively since the first demonstration in 2007 due to the distinguishing properties of nondiffraction, transverse acceleration and self-healing. To date, a simple and compact approach for generating Airy beams in high quality and efficiency has remained challenging. Here, we propose and demonstrate a liquid crystal (LC) polarization Airy mask (PAM) featured by spatially variant LC azimuthal director. The PAM is fabricated through photoaligning LC via a polarization-sensitive alignment agent suophonic azo dye SD1. Thanks to the special design, a novel feature of polarization-controllable switch between dual Airy beams of orthogonal circular polarization is presented. The molecular-level continuity of LC director significantly improves the quality and efficiency of resultant Airy beams. Besides, the PAM can handle intense light due to the absence of absorptive electrodes. Additional merits of compact size, low cost and broad wavelength tolerance are also exhibited. This work settles a fundamental requirement for Airy beam applications of optical manipulations, biology science and even some uncharted territories.

The last few years have witnessed a rapid growth in the research of Airy beams. An ideal Airy beam is infinite in space and energy. Till 2007, an exponential aperture was introduced to demonstrate Airy beams in practice^1^. They still retain the distinguishing features of nondiffraction, transverse acceleration and self-healing^2^,^3^, inspiring broad explorations. The nondiffraction nature motivates Airy-Bessel light bullets^4^. The transverse acceleration property facilitates the generation of curved channels^5^,^6^, as well as the micromachining of curved profiles^7^. Thanks to the self-reconstructing^8^, Airy beams have been utilized in light-sheet microscopy for high contrast over an extended field of view^9^,^10^ and free-space optical communication against atmospheric turbulence^11^. Moreover, Airy beams can also realize clearing and redistribution of microspheres or cells^12^,^13^, and be applied in nonlinear optics^14^,^15^, plasmonics^16^,^17^,^18^, electronics^19^ etc.

To date, several techniques have been developed to generate finite Airy beams based on cubic phase modulations of Gaussian beams. A straightforward way is employing a cylindrical lens of specific curvature radius^20^ or tilted cylindrical lenses system^21^ for directly rephasing. Nevertheless, the elements are bulky and the technique suffers from custom design, complicated manufaction or precise alignment. To compact the element, a photoresist mask with a cubic phase modulo 2π was applied^5^,^22^. Whereas, a delicate laser direct writing lithography is required. A similar “mask” could also be output by a spatial light modulator (SLM)^1^,^23^. However, the SLM consists of numerous discrete micro-size pixels driven by complex electrode matrix^24^, making the strategy costly and optically inefficient (~40%)^22^, and limiting the quality of output beams. To simplify the configuration, polymer-dispersed liquid crystals (LCs)^25^ and patterned electrodes^26^,^27^ have been introduced to execute binary cubic phase patterns. Unfortunately, in these cases, two Airy beams appeared simultaneously, restricting their applications. Hence, exploring new simple and compact approaches for generating single Airy beam in high quality and efficiency is in urgent demand.

Above LC techniques are based on the phase modulation through electrically tuning the tilt angle of LC. In fact, the azimuthal angle could be controlled to realize above modulation as well. It can be conveniently accomplished by photoalignment, which supplies a suitable approach for high-resolution LC alignment in displays^28^ and other optics fields^29^,^30^. In this work, by means of a polarization-sensitive alignment agent and a digital micro-mirror device (DMD) based microlithography system, a LC polarization Airy mask (PAM) with space-variant azimuthal orientations is demonstrated. The principle here is quite similar to the recently reported Airy beam converter, which is fabricated with femtosecond laser-imprinted sub-wavelength nanoplanes in silica glass^31^. The design presented merits of polarization-controllable switch between single and dual Airy beams and resistance to intense light. However, the fabrication is still costly and time consuming, and the converter works only for a fixed wavelength. Our proposed approach supplies high-quality Airy beam generation avoiding above problems, and it may open a door towards widespread applications even in some unexplored fields.

## Results

### Design and fabrication of the PAM

**Figure 1:**
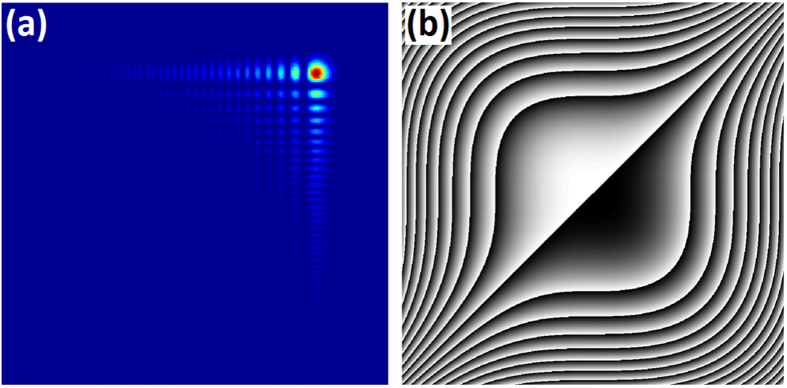
2D Airy beam and phase pattern. (**a**) Simulated intensity distribution of a 2D Airy beam with *a* = 0.04. (**b**) The cubic phase pattern wrapped between 0 and 2π, where black to white indicates 0 to 2π.

The envelope of a finite Airy beam is expressed as^1^:


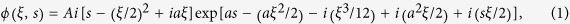


where *Ai*(**∙**) represents Airy function^32^, *a* is a positive parameter and *a*«1, *s* = *x/x*_*0*_ is a dimensionless transverse coordinate, *x*_*0*_ is an arbitrary transverse scale, 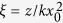 is the normalized propagation distance, and *k* = 2π*n/λ*_*0*_ is the wavenumber of the optical wave with index of refraction *n* and wavelength *λ*_*0*_ in vacuum. The envelope is described by an Airy function centered on a quadratic parabolic trajectory and a typical intensity distribution of a two-dimensional (2D) Airy beam with *a* = 0.04 is illustrated in Fig. 1a. It consists of a main lobe and a family of side beamlets whose intensity decay exponentially. The Fourier transform of the envelope is


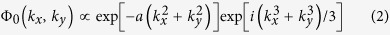


for a 2D case, where *k*_*x*_ and *k*_*y*_ are Fourier spectrum coordinates^1^. That means an Airy beam can be treated as a Gaussian beam modulated by a cubic phase. Figure 1b shows the phase pattern of a 2D Airy wave packet with the phase range varying from −15π to +15π. An Airy mask thus could be obtained by inscribing the phase pattern into certain medium.

**Figure 2:**
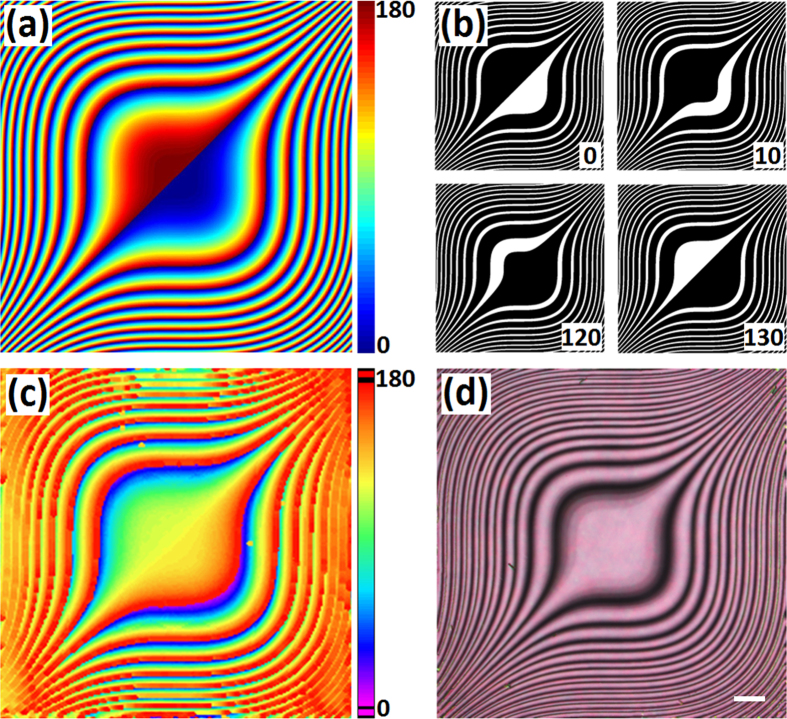
Fabrication and characterization of PAM. (**a**) Optical axes orientation of a PAM. The color variation from blue to red indicates the optical axis varying from 0° to 180°. (**b**) Four examples of exposure patterns with corresponding polarizer angles labelled in the corner. (**c**) The measured azimuthal director distribution of the LC PAM. The color bar indicates the director orientation. (**d**) Micrograph of the same sample. The scale bar is 100 μm.

Here we introduce a similar concept of polarization gratings^33^,^34^ to Airy masks, namely polarization Airy mask. Each region from 0 to 2π in Fig. 1b is replaced by a continuous optical axes orientation from 0° to 180° according to *φ*(*x*, *y*) = *x*^3^ + *y*^3^ in the *x–y* plane, as shown in Fig. 2a. To accomplish such a PAM, a polarization-sensitive photoalignment agent sulphonic azo-dye SD1 is adopted. Under UV exposure, the dye molecules tend to reorient their absorption oscillators perpendicular to the incident polarization^35^,^36^,^37^. The orientation of SD1 will spread to adjacent LC molecules thus guiding their local azimuthal directors. A DMD based micro-lithography system^38^ is employed to transfer the pattern into LC cells. After an eighteen-step five-time-partly-overlapping exposure^39^, a quasi-continuous space-variant orientation of SD1 is carried out. Four exposure patterns with corresponding polarizer angles are given as examples in Fig. 2b. When LC E7 is capillarily filled, a director variant LC PAM forms owing to the excellent fluidity and continuity of LC.

Figure 2c reveals the real azimuthal director distribution detected via a two-dimensional Stokes parameters measurement. As expected, a 90° shift is observed compared to Fig. 2a, because the SD1 molecules orientate perpendicularly to the incident polarization. Due to the resolution limitation of the measurement setup, details of the director distributions are not faithfully presented. Actually, the LC directors vary continuously and periodically, which can be further proved by observations under a polarized optical microscope. As shown in Fig. 2d, the micrograph provides a more vivid exhibition of the PAM with the brightness varying continuously. The bright regions indicate the LC directors are around 45° with respect to the polarizer, whereas the dark regions indicate the LC directors are about parallel to the polarizer^40^. When rotating the sample under the microscope, the dark and bright regions interconvert consecutively, confirming the continuous and periodic orientation of LC. This reveals that the pattern of the designed PAM has been accurately transferred into a LC cell and the alignment has been precisely controlled.

### Polarization-controllable Airy beams

**Figure 3:**
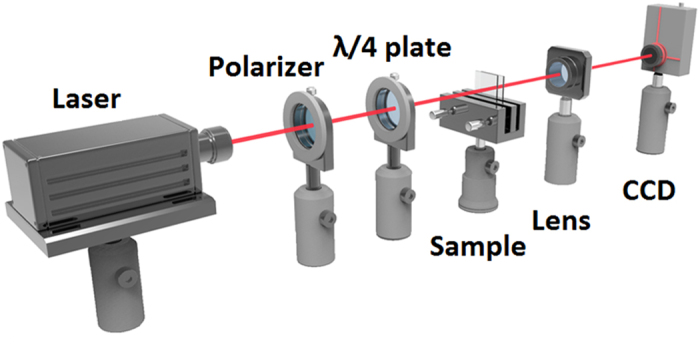
Experimental setup. A scheme of the optical setup for Airy beam reconstruction.

To characterize the performance of the PAM, several tests have been carried out. Figure 3 illustrates the optical setup for Airy beam reconstruction. A Gaussian beam from a 671 nm laser propagates through a polarizer, a λ/4 plate, the sample, a spherical lens (*f* = 125 mm) in sequence, and then the resultant Airy beam is captured by a charge coupled device (CCD) camera. The λ/4 plate is rotated to tune the angle (*θ*) between its c-axis and the polarizer direction, thus adjusting the incident polarization. The lens is set at a distance of *f* behind the sample to perform the Fourier transformation. Herein, we defined the focal plane of the lens as the original observation point (*d* = 0) and the finite Airy beams were recorded at a distance of *d* ≥ 0.

In our work, the phase retardation is fixed at 3π @ 671 nm. Jones calculus can be used for calculating the amount of phase added to the polarized light by the λ/2 plate, which stands for the PAM in this situation. Multiplying λ/2 plate Jones matrix M with Jones vector for the right circular polarization, we obtain^31^


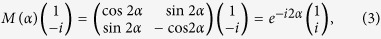


where α is the angle of the λ/2 plate slow axis. The first term shows the phase delay that depends on the spatial optical axis orientation of λ/2 plate (φ = 2α). The second term of the product indicates that the handedness of polarization flips from right to left, vice versa. Thus, the azimuth of the λ/2 plate slow axis is modulated as:


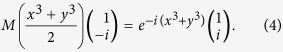


As a result, a PAM is implemented, with the output diffraction controlled by the polarization of the incident light.

**Figure 4:**
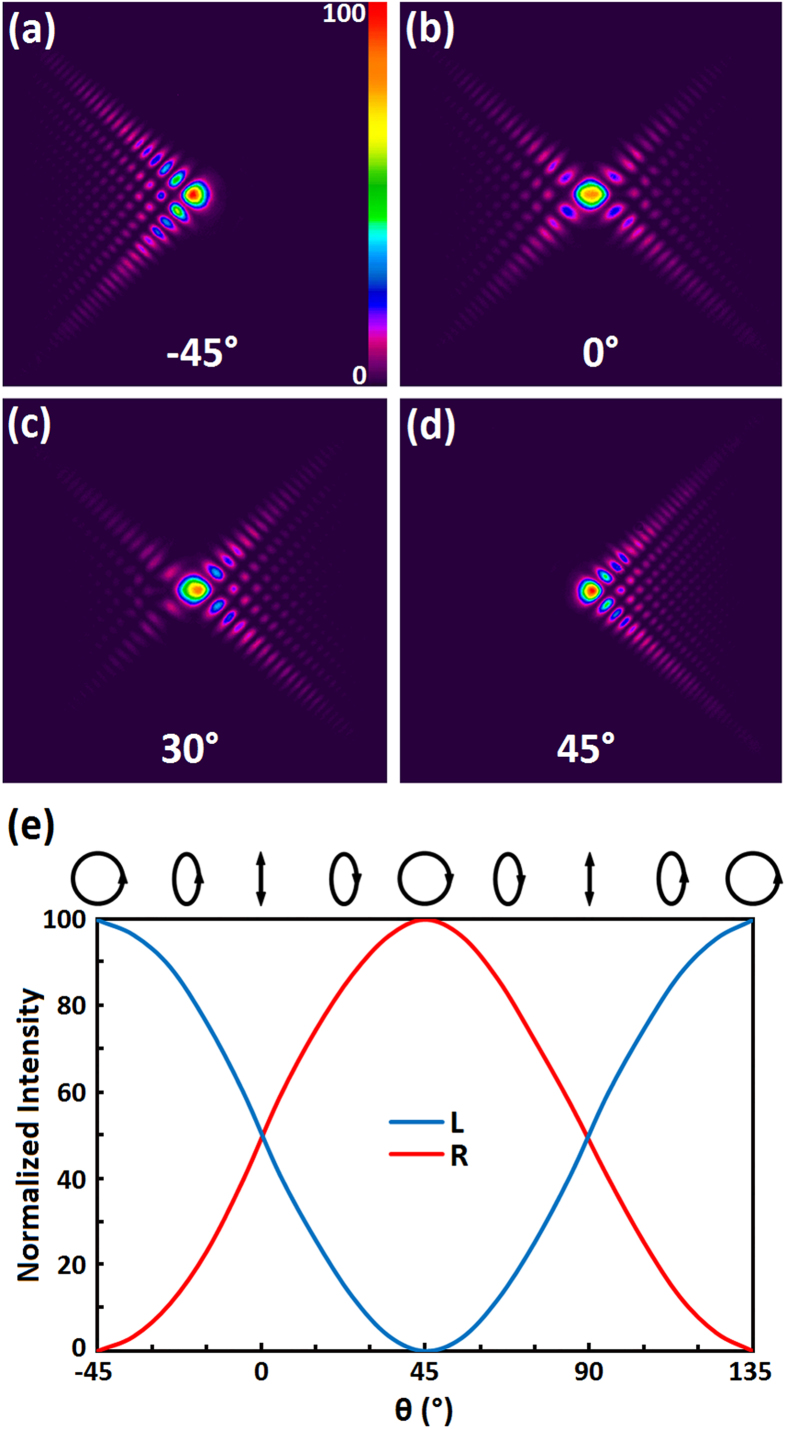
Polarization-controllable Airy beams. Images of Airy beams illuminated by (**a**) right circularly (**b**) linearly (**c**) elliptically and (**d**) left circularly polarized light, respectively. The angle between the c-axis of λ/4 plate and the polarizer direction is labelled correspondingly. The color bar indicates the relative optical intensity. (**e**) The dependency of normalized intensity on incident polarization. The blue curve stands for the left branch, while the red curve stands for the right one.

Some images of Airy beams are presented in Fig. 4 taken at *d* = 10 cm. Figure 4a depicts the generated Airy beams when *θ* = −45°, i.e., right circularly polarized light illuminated. Only one Airy beam was observed, exhibiting an orthorganal polarization to incident light. When illuminated by left circularly polarized light, only a converse Airy beam appeared, as shown in Fig. 4d. Dual orthorganally polarized Airy beams occurred under the condition of linearly polarized illumination, as revealed in Fig. 4b when *θ* = 0°. Along with the tuning of *θ*, the energy distribution between the dual branches can be continuously modulated. Figure 4c gives an example when *θ* = 30°. Figure 4e plots the experimental results of *θ* dependent normalized intensity of the separate branches, denoting a strong and opposite evolution on the incident polarization. Above phenomena are quite consistent with the analysis above, permitting a novel feature of polarization control switch to Airy beams.

Comparing Fig. 4a with Fig. 1a, the obtained Airy beam is perfectly consistent with the simulation, and a more vivid comparison could be observed in three-dimensional images (see supplementary Fig. S1). The high quality benefits from our novel design. The resolution of the DMD based lithography system reaches 1 μm and the final LC director distribution is continuous at the molecular level. Thereby, it ensures a reliable and smooth pattern transfer, guaranteeing high quality of the resultant Airy beams which could hardly be achieved by the pixelated SLM, binary LC elements and other previous techniques. Moreover, the Airy beams exhibit characteristic of polarization controllable energy distribution between two orthogonal circularly polarized branches. This gives extra advantages which may promote considerable applications, such as chirality selective optical manipulations^41^. The SD1 is compatible to the azimuthal angle control of most LCs. For a given wavelength (visible, infrared to terahertz)^42^,^43^, we can properly choose LC materials and precisely control the cell gap to optimize the conversion efficiency. In addition, thanks to the excellent electro-optical tunability of LCs, good tolerance to incident light wavelength and electrical switching between Airy beams and Gaussian beams are achievable via tuning the applied voltage. In our experiment, incident laser beams with wavelength ranging from 432 nm to 722 nm were converted to Airy beams high efficiently (see supplementary Fig. S2). Furthermore, the minification and magnification can be easily tuned by adjusting the objective in the DMD based system^38^, making the final PAM size perfectly match the incident beam. As the PAM here is induced by azimuthal angle control of LC (see supplementary Fig. S3), absorptive electrodes are avoided, therefore the optical damage threshold will be drastically increased. In our preliminary trials, no damage was observed after 600 pulses (0.5 J/cm^2^, 1064 nm, 10 ns, 1 Hz) illumination. The value is an order higher than the damage threshold of commercial SLM at the same conditions. That means the sample is promising for intense light applications such as light bullets^4^. Due to the limitation of our equipment, the exact damage threshold could not be obtained at present and further research is on the way.

### Transverse acceleration and nondiffraction

**Figure 5:**
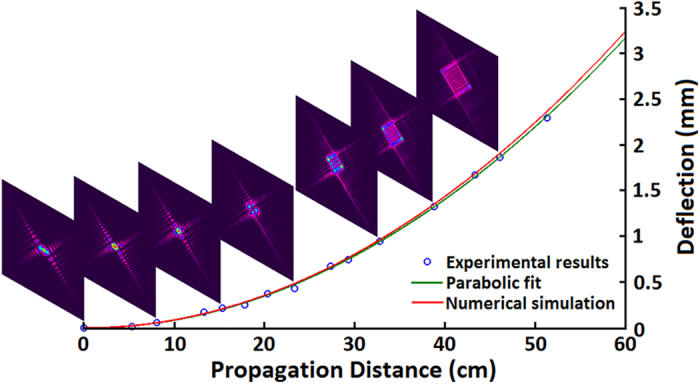
Transverse acceleration. Transverse acceleration of Airy beam as a function of propagation distance. Blue circles mark the experimental results with some images inserted accordingly. The green line is the parabolic fit while the red line is the numerical simulation.

To verify the features of generated Airy beams, transverse acceleration and nondiffraction properties were investigated. Cases of dual Airy beams were analyzed, as the transverse deflection (*x*_*d*_) of the Airy beams could be directly obtained from the distance between the two main lobes (*x*_*D*_), i.e., *x*_*d*_ = *x*_*D*_/2, avoiding the deviation involved by the move of CCD. The transverse deflections at certain propagation distances were measured and corresponding results are marked as blue circles in Fig. 5. The transverse profiles at several propagation distances are inserted, vividly showing the beam deflection along propogation. The green line, which is a parabolic fit of the relationship between *x_d_* and *d*, matches the simulation red curve well. That proves a remarkable transverse acceleration. From these images, we can also see that the beam profile of each single branch does not change along propagation. The phenomenon verifies the nondiffracting property.

## Discussion

We have demonstrated a LC PAM by means of a polarization-sensitive alignment agent SD1 and a DMD based microlithography system. Through a multi-step and partly-overlapping exposure strategy, the designed pattern is accurately transferred and continuous variation of LC azimuthal director is obtained. The proposed design brings new extraordinary characteristics and significantly improves the performance of generated Airy beams. Polarization-controllable switch between dual Airy beams of orthogonal circular polarization has been firstly demonstrated in LC based elements. The Airy beams are demonstrated up to an unprecedented quality, and their nondiffraction and transverse acceleration features are well verified. The PAM can handle intense light due to the absence of absorptive electrodes. Besides, the LC PAM exhibits merits of campact size, low cost, broad wavelength tolerance and good electrical tunability. This work settles a fundamental requirement for Airy beam applications, which has great potentials in the fields of optical manipulations, polarization imaging, micro-fabrications, biology science and even some unchartered territories.

## Methods

### Chemicals and reagents

Fused quartz substrates were ultrasonic bathed, UV-Ozone cleaned and then spin-coated with 0.5% solution of sulphonic azo-dye SD1 (Dai-Nippon Ink and Chemicals, Japan) in dimethylformamide (DMF). The cell was infiltrated with LC mixture E7.

### Cell assembling and photoalignments

4.5 μm spacers were spurted over one substrate then the counter substrate was put over it. The assembled cell was sealed by epoxy glue. Afterwards the cell was placed at the image plane of the DMD based microlithography system to record the designed patterns. The image of optical axes orientation was devided into eighteen sub-regions with equal angle range of π/18. Each sub-region was endowed with a uniform value, from 0 to 17π/18 in intervals of π/18. A sum of five adjacent sub-regions (i.e., the sum-region) was exposed simultaneously with an exposure dose of 1 J/cm^2^. The subsequent exposure of the sum-region shifted one sub-region with the polarizer rotating 10° synchronously. After the eighteen-step five-time-partly-overlapping exposure with a total exposure dose of 5 J/cm^2^, a quasi-continuous space-variant orientation of SD1 was carried out. After the LC was capillarily filled, the director variant LC PAM was formed.

### Director distribution characterizations

The setup for two-dimensional Stokes parameters measurement consists of a polarizer, a λ/4 plate, a holder for samples, another λ/4 plate and a polarizer mounted on motorized rotators in sequence. A CCD is used as a two-dimensional detector array for the simultaneous detection of all four Stokes parameters of the output optical image. A LabVIEW program is used to control the two rotators, as well as to record and calculate the data.

## Additional Information

**How to cite this article**: Wei, B.-Y. *et al.* Polarization-controllable Airy beams generated via a photoaligned director-variant liquid crystal mask. *Sci. Rep.* **5**, 17484; doi: 10.1038/srep17484 (2015).

## Supplementary Material

Supplementary Information
