# Supplementary material for: Polarization-controllable Airy beams generated via a photoaligned director-variant liquid crystal mask
Source: Sci Rep. 2015 Dec 2;5:17484. doi: 10.1038/srep17484 (PMC4667248; doi:10.1038/srep17484)
Supplement: Supplementary Information [file srep17484-s1.pdf]

Polarization-controllable Airy beams generated via a photoaligned director-variant liquid crystal mask

Bing-Yan Wei[1], Peng Chen[1], Wei Hu[1,*], Wei Ji[1], Li-Yang Zheng[1], Shi-Jun Ge[1], Yang Ming[1], Vladimir Chigrinov[2] & Yan-Qing Lu[1,*]

[1] National Laboratory of Solid State Microstructures, Collaborative Innovation Center of Advanced Microstructures and College of Engineering and Applied Sciences, Nanjing University, Nanjing 210093, China.

[2] Center for Display Research, Department of Electronic and Computer Engineering, Hong Kong University of Science and Technology, Clear Water Bay, Kowloon, Hong Kong, China.

[*]Correspondence and requests for materials should be addressed to W. H. (email: huwei@nju.edu.cn) and to Y. L. (email: yqlu@nju.edu.cn).

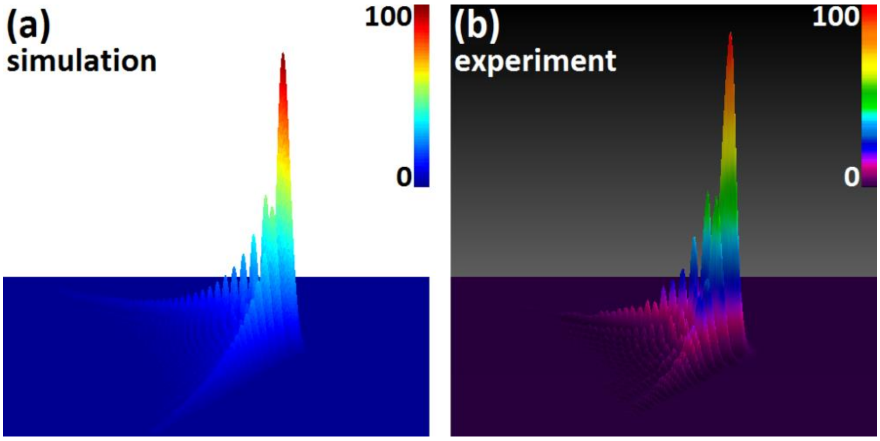

Figure S1. 3D intensity distributions of (a) simulated and (b) experimentally obtained Airy beams.

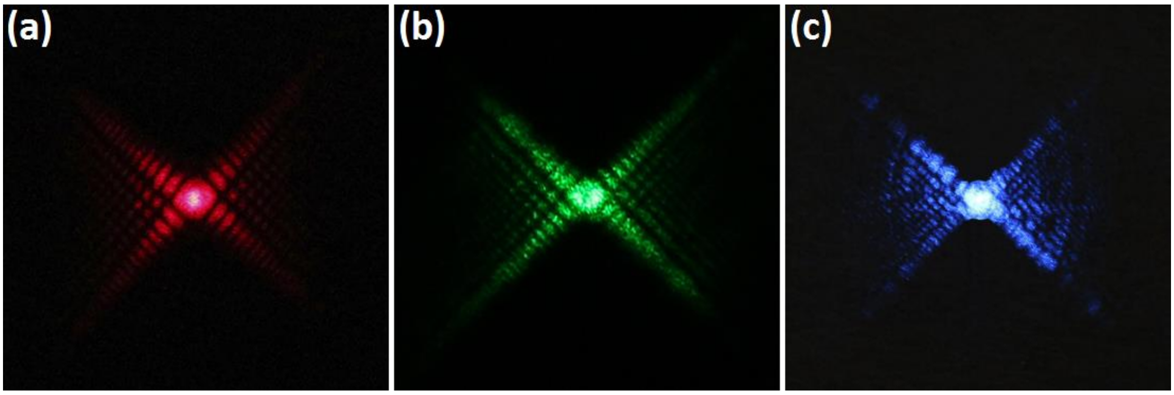

Figure S2. Airy beams of different wavelengths: (a) 722 nm, (b) 532 nm and (c) 473 nm.

The Airy beams in different colors captured by camera proved that the PAM can work at a wide spectrum via applying proper voltages. Due to the lack of corresponding quarter waveplates, Airy beams with two branches are exhibited. According to the equation[1] $2\pi\Delta nd/\lambda = \pi$ (taking $\Delta n = 0.2$, $d = 4.5$ μm), our sample could work at the maximum wavelength of 1.8 μm. The value could be further increased by introducing larger d. As the SD1 absorbs 473 nm laser light, the pattern decays after long time illumination. Therefore, the profile of blue Airy beams is not so good as red and green ones.

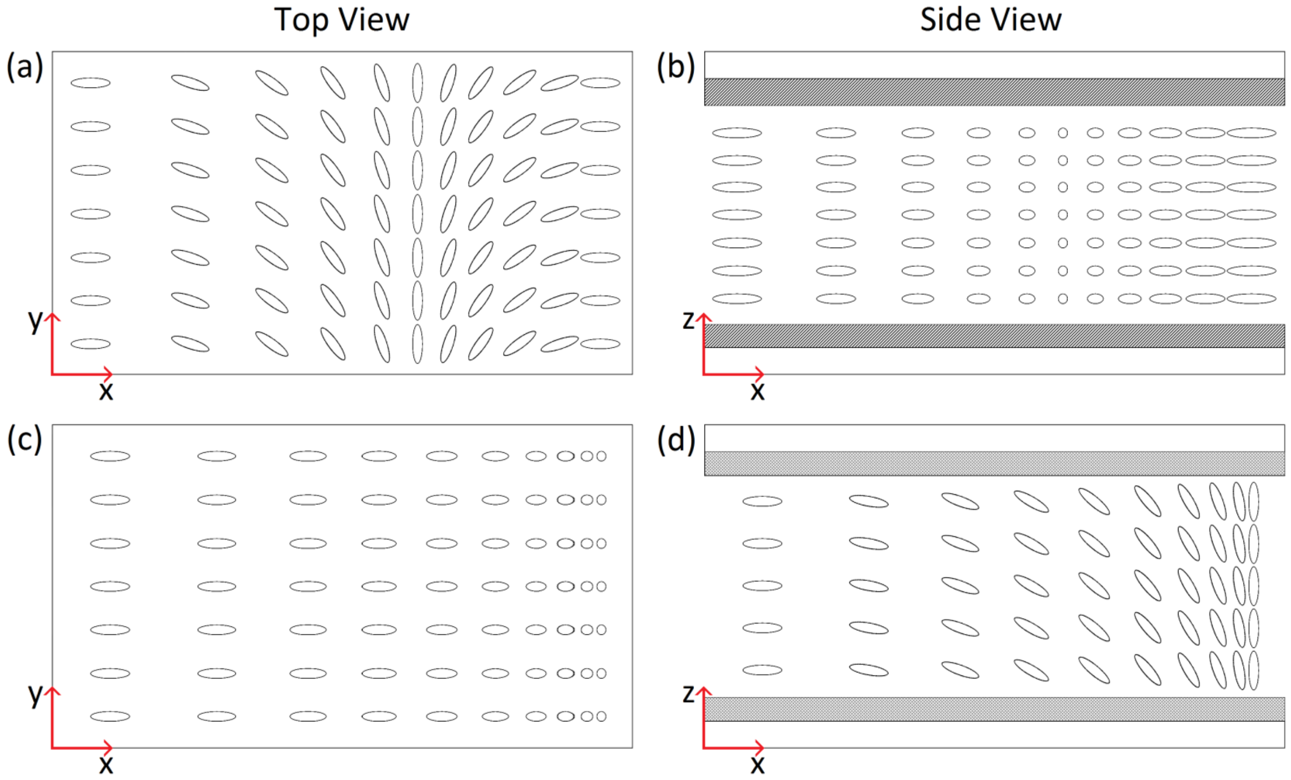

Figure S3. Diagrams of the LC director orientation of the proposed PAM and commercial SLM. (a) top view and (b) side view of PAM ; (c) top view and (d) side view of SLM.

Figure S3 schematically reveals the different LC director orientations of the PAM and SLM. We take a single period of the cubic phase pattern as an example, therefore the orientation of LC directors changes nonlinearly along the *x* direction. Figure S3a and b are the top and side views of PAM respectively, while c and d are those of SLM. For PAM, the azimuthal angles of LCs are arranged from 0 to π in the *x*-*y* plane and the tilt angle is fixed 0 in the *x*-z plane. While in commercial SLM, the cubic phase retardance is realized by electrically tuning the tilt angle of LC molecules (0 to π/2 for instance here). According to the equation[1]

$$n_{eff}(z) = \frac{n_e n_o}{\sqrt{n_e^2 \sin^2 \theta(z) + n_o^2 \cos^2 \theta(z)}}, \quad \text{(S1)}$$

where n_{eff} is the effective refractive index of LC, $\theta(z)$ is the tilt angle defined as the angle between LC director and *x*-*y* plane. When different electric fields in different areas are applied along the *z* direction, the LC molecules tilt at different angles and the light will encounter the corresponding refractive index n_{eff}. Therefore the cubic retardation of the LC is formed.

Supplementary Reference:

1. Yang, D. K. & Wu, S. T. *Fundamentals of liquid crystal devices*. (Wiley, England, 2006).
